# Electrocardiographie couplé à l’échocardiographie transthoracique de repos dans le diagnostic des atteintes cardiaques chez le diabétique de type 2: les enseignements d’une série transversale au Burkina Faso

**DOI:** 10.11604/pamj.2018.31.169.15798

**Published:** 2018-11-09

**Authors:** Somnoma Jean-Baptiste Tougouma, Yibar Kambiré, Jonas Bado, Aimé Arsène Yaméogo, Téné Marceline Yaméogo, Samba Sidibé, Carole Gilberte Kyelem, Alassane Ilboudo, Macaire Ouédraogo

**Affiliations:** 1 Institut Supérieur des Sciences de la Santé (INSSA), Université Nazi Boni de Bobo-Dioulasso (UNB), Burkina Faso; 2 Unité de Formation et de Recherche en Sciences de la Santé, Université de Ouagadougou, Burkina Faso; 3 Centre Hospitalier Universitaire Sourô Sanou (CHUSS), Bobo-Dioulasso, Burkina Faso; 4 Centre Hospitalier Universitaire du Point G, Bamako, Mali

**Keywords:** Diabète de type 2, électrocardiographie, échocardiographie, Burkina Faso, Type 2 diabetes, electrocardiography, echocardiography, Burkina Faso

## Abstract

Le diabète est un puissant facteur de risque cardio-vasculaire indépendant. Les auteurs se proposent de décrire les anomalies électrocardiographiques et échocardiographiques observées chez les diabétiques de type 2 suivis dans le département de médecine du CHUSS. Une étude transversale descriptive a été réalisée d’avril à septembre 2014 incluant tous les diabétiques de type 2 consentants. Tous ont bénéficié d’un recueil de données cliniques, d’une électrocardiographie et d’une échocardiographie doppler. Un total de 155 diabétiques a été enquêté. L’âge médian de la population était de 55 ans (IIQ: 47-64) à prédominance féminine (Sex ratio 0,5). Les anomalies électrocardiographiques étaient représentées par les troubles de la repolarisation (31%) et les troubles du rythme auriculaire (16,12%). Sur le plan échocardiographique, l’hypertrophie du ventricule gauche (VG) était rapportée dans 20,64%. L’oreillette gauche était dilatée dans 14,19%, une dilatation VG dans 1,3% des cas. Une altération de la fraction d’éjection VG était notée dans 3,87% des cas. Les entités nosologiques étaient représentées par la cardiomyopathie hypertensive dans 27 cas (54%), la cardiopathie ischémique dans 19 cas (38%), la cardiomyopathie dilatée dans 2 cas (4%) et la cardiomyopathie diabétique dans 2 cas (4%). L’insuffisance cardiaque était notée dans 22 cas (44%) indépendamment de l’atteinte cardiaque présentée. Les anomalies électrocardiographiques et échocardiographiques sont fréquentes dans la population de diabétique de type 2 au CHUSS de Bobo-Dioulasso. Une meilleure collaboration entre cardiologues et diabétologues et la mise en place d’un plateau technique adéquat de dépistage serait un préalable pour une meilleure stratification du risque cardiaque dans cette population.

## Abstract (en)

Diabetes is a powerful independent cardiovascular risk factor. The aim of this study is to describe the electrocardiographic and echocardiographic abnormalities observed in patients with type 2 diabetes treated in the Department of Medicine at the University Hospital in Bobo-Dioulasso. We conducted a descriptive cross-sectional study of all patients with type 2 diabetes who gave consent from April to September 2014. We gathered clinical data from all the patients. They, moreover, underwent electrocardiography and doppler echocardiography. A total of 155 diabetics were investigated. The average age of patients was 55 years (IQR: 47-64) with a female predominance (sex ratio 0.5). Electrocardiographic abnormalities included repolarization abnormalities (31%) and atrial rhythm disorders (16,12%). Echocardiographic examination showed left ventricular hypertrophy (LVH) in 20,64% of cases. Left atrium was dilated in 14.19% of cases, LV was dilated in 1.3% of cases. Abnormal left ventricular ejection fraction was detected in 3.87% of cases. Nosological entities included hypertensive heart disease in 27 cases (54%), ischemic heart disease in 19 cases (38%), dilated cardiomyopathy in 2 cases (4%) and diabetic cardiomyopathy in 2 cases (4%). Heart failure was detected in 22 cases (44%) independently from cardiac impairment. Electrocardiographic and echocardiographic abnormalities are frequent in type 2 diabetes population at the University Hospital in Bobo-Dioulasso. Improved cooperation between cardiologists and diabetologists as well as the establishment of adequate technical screening equipment would be prerequisite for better cardiac risk stratification in this population.

## Introduction

Depuis le début des années 2000, la plupart des pays africains subsahariens connaissent une augmentation des phénomènes morbides chroniques non transmissibles cardio métaboliques qui coexistent avec les maladies transmissibles infectieuses, constituant ainsi le double fardeau des pays africains subsahariens. Le Burkina Faso, non en marge de ce double fardeau, connait une hausse progressive des cas de diabète sucré avec une prévalence rapportée en 2013 de 4,9% [1]. Le diabète est un puissant facteur de risque cardio-vasculaire indépendant [2]. La première cause de décès chez les diabétiques est due aux atteintes cardio-vasculaires [3, 4]. Cette lourde mortalité attribuable aux atteintes cardiaques est bien souvent d’installation insidieuse et parfois asymptomatique comme la maladie coronaire et l’insuffisance cardiaque. Leur diagnostic fait appel à des examens complémentaires dont les plus simples demeurent l’électrocardiographie et l’échocardiographie. Au Burkina Faso, peu d’études ont évalué les caractéristiques électrocardiographiques et échocardiographiques au cours du diabète de type 2 [5] et plus particulièrement à l’électrocardiographie (CHUSS) de Bobo-Dioulasso où aucun travail n’a encore été réalisé sur le sujet. Nous nous sommes fixés pour objectif de décrire les caractéristiques électrocardiographiques et échocardiographiques et d’en définir les entités pathologiques qui en découlent dans une population de diabétique de type 2 suivis dans le Département de Médecine du CHUSS.

## Méthodes

**Type d’étude, période d’étude critères d’inclusion:** Il s’est agi d’une étude descriptive transversale réalisée d’avril à septembre 2014 dans le Département de Médecine du CHUSS de Bobo-Dioulasso.

**Population d’étude et critères d’inclusion:** Il s’agissait des patients diabétiques de type 2 des deux sexes, suivis en ambulatoire dans le Département de Médecine du CHUSS et consentant à participer à l’étude.

### Méthodes et techniques de collecte des données

Les patients diabétiques ont été mobilisés pour l’étude via les médecins internistes et cardiologues au cours des consultations de suivi. Les patients consentants étaient accueillis dans le Service de Cardiologie du CHUSS dans une salle réservée à cet effet pour le processus d’information et de consentement éclairé pour la participation à l’étude. Après la signature du consentement, chaque enquêté bénéficiait d’un interrogatoire et d’un examen clinique complet notamment cardiovasculaire; d’un enregistrement électrocardiographique de repos (12 dérivations) à l’aide d’un appareil de marque HELLIGE EK53. Il bénéficiait également d’une échographie Doppler cardiaque transthoracique (ETT) de repos à l’aide d’un appareil de marque ALOKA PROSOUND 400 doté de fonctionnalités Doppler couleur, pulsé et tissulaire, mis en service en 2010. Une prise de sang après 12 heures de jeûn a été réalisée chez l’ensemble des patients inclus. Cette prise de sang a permis le dosage de la glycémie, l’hémoglobine glyquée et un dosage complet des lipides.

A l’interrogatoire les données collectées étaient: les caractéristiques sociodémographiques (âge, sexe, profession, niveau d’étude), les données sur le diabète (ancienneté, traitement, complications…), les facteurs de risque cardiovasculaire: hypertension artérielle (HTA) personnelle ou familiale, surpoids, tabagisme actif ou passif, alcoolisme, dyslipidémie; l’hérédité cardiovasculaire (HTA, coronaropathie/accident ou maladie cardiaque et antécédent de mort subite familiale avant 50 ans). Les données collectées lors de l’examen clinique étaient: les constantes anthropométriques et cliniques (poids, taille, indice de masse corporelle, surface corporelle, fréquence cardiaque, pouls, pression artérielle après 15 minutes de repos); les signes cliniques (douleur angineuse, palpitations, dyspnée d’effort, rythme cardiaque, souffles cardiaques, pouls pédieux).

A l’ECG les variables suivantes étaient recueillies: fréquence cardiaque de repos, rythme, durée et amplitude de l’onde P, durée de l’espace PR, durée du complexe QRS, l’axe des QRS, index de Sokolov-Lyon, le QT corrigé ainsi que l’aspect de la repolarisation. A l’échocardiographie Doppler Transthoracique de repos, les paramètres suivants ont été recueillis: le diamètre télédiastolique du ventricule gauche (DTDVG) ainsi que le DTDVG indexé, la fraction d’éjection du ventricule gauche (FEVG), les épaisseurs télédiastoliques du Septum Inter Ventriculaire (SIVd) et de la Paroi Postérieure (PPd) du ventricule gauche, le volume télédiastolique de l’oreillette gauche (en 4 et 2 cavités), le calcul de l’épaisseur pariétale relative (h/r) et de la masse ventriculaire gauche. Les paramètres doppler pulsé et tissulaire ont été utilisés pour l’évaluation de la fonction diastolique du ventricule gauche (Em/Am, Em/E’, Ap-Am) et du ventricule droit (l’excursion systolique du plan de l’anneau tricuspide (TAPSE) et onde Sa à l’anneau). Les résultats des enregistrements électrocardiographiques étaient validés par deux cardiologues. Les images échocardiographiques étaient recueillies de façon standardisées et archivées dans l’appareil et les anomalies étaient visionnées et validées par le staff d’échocardiographie. Un prélèvement sanguin était effectué au lendemain de l’interrogatoire et de l’examen clinique après 12h de jeûn pour le dosage de la glycémie, l’hémoglobine glyquée, le cholestérol total, le HDL-cholestérol, LDL-cholestérol et les triglycérides.

### Taille de l’échantillon

L’échantillonnage a tenu compte de l’ensemble des patients diabétiques de type 2 dans la file active, suivis dans le Département de Médecine à cette date. Nous avons utilisé OPENEPI [6] pour calculer la taille minimale de l’échantillon. La prévalence attendue de l’ischémie myocardique silencieuse dans la population d’étude fixée à 16,6%; la précision souhaitée à 5% et z 1-a/2 = 1.96. A partir de la file active de patients diabétique de type 2, la population source a été estimée à 300 sur la base des statistiques annuelles du Service de la Documentation et de l’Information Médicale du CHUSS. Cela donne une taille minimale de 123.

### Le traitement des variables

L’HTA était définie par une PAS ≥ 140mmHg et/ou PAD ≥ 90mmHg et/ou sous traitement antihypertenseur quel que soit la régularité du traitement; l’ischémie sous épicardique était définie par la présence d’ondes T négatives concordantes dans un territoire coronaire; la lésion sous endocardique était définie par un sous décalage significatif du segment ST (> 1,5mm) concordant dans un territoire coronaire; la séquelle de nécrose était définie par une abrasion des ondes R concordantes dans un territoire coronaire et/ou une onde Q large (≥ 4/100^ème^ sec) et profonde (≥ 1/10^ème^ mv). L’hypertrophie ventriculaire gauche (HVG) était définie par un indice de Sokolov-Lyon ≥ 35mm. L’allongement de l’espace QT était défini par un QT calculé selon la formule de Bazett = 0,46s. La dilatation du VG était définie par un diamètre télédiastolique du ventricule gauche > 31mm/m^2^ chez l’homme et > 32mm/m^2^ chez la femme [7].

L’hypertrophie ventriculaire gauche (HVG) à l’ETT était définie par une masse ventriculaire gauche (MVG) indexée ≥ 95g/m^2^ chez la femme et 115g/m^2^ chez l’homme [8]; l’évaluation de la géométrie du VG était basée sur la valeur de l’épaisseur relative (h/r) définie par le rapport: épaisseur septum inter-VG en diastole (SIVd) + épaisseur paroi postérieure en diastole (PPd)/diamètre télédiastolique VG (DTDVG). La valeur normale est < 0,44 [8]; l’évaluation des pressions de remplissage du ventricule gauche (PRVG) était définie suivant les recommandations américaines et celles européennes [9]; l’oreillette gauche (OG) dilatée était définie par un volume > 35ml/m^2^ mesuré en deux et quatre cavités à l’ETT [8]; le remodelage VG était défini par une MVG indexée normale et une h/r > 0,44 [8].

La cardiomyopathie diabétique était définie comme une dysfonction diastolique avec conservation de la fonction systolique chez le diabétique non hypertendu, sans antécédents de coronaropathie, ni de valvulopathie. La cardiopathie ischémique était définie par la conjugaison de plusieurs facteurs cliniques (douleurs d’allure angineuse) électrocardiographiques (signe d’ischémie ou de nécrose concordant dans un territoire coronaire) et échocardiographique (dyskinésie, hypokinésie ou akinésie segmentaire). La cardiomyopathie hypertensive était définie par une HTA associée à une atteinte structurelle par HVG à l’échocardiographie. L’insuffisance cardiaque était définie par une élévation des pressions de remplissage du VG associées ou non à une altération de la fonction systolique du ventricule gauche.

### Analyse des données

Les données recueillies sur la fiche technique ont été saisies et analysées à l’aide des logiciels Epi Data version 3.1 et Stata version 12.0. L’analyse a consisté à la production de statistiques descriptives (médiane ou proportion) de la population d’étude.

### Considérations éthiques

La participation à notre étude était conditionnée par la signature d’un consentement éclairé. Le refus de prendre part à l’étude n’entrainait aucun préjudice. La confidentialité a été respectée et le traitement des données a été anonyme. La participation à l’étude n’entrainait aucun frais pour les sujets inclus.

## Résultats

### Caractéristiques de la population

Cent cinquante-cinq patients ont été évalués. L’âge médian de la population était de 55 ans (IIQ: 47-64), à prédominance féminine (sexe ratio de 0,50). Les facteurs de risque cardiovasculaire retrouvés en dehors du diabète sucré étaient les anomalies lipidiques dans 62 cas (40%), le tabagisme actif dans 13 cas (8,39%), l’obésité dans 105 cas (67,77%) et l’alcool dans 22 cas (14,19%). L´HTA était retrouvée dans 104 cas (67,9%) dont 68,27% (71/104) étaient hypertendus connus. L’HTA était insuffisamment traitée dans 54 cas (76,5%). La pratique d’une activité physique régulière était retrouvée chez 32 patients (20,65%). Il s’agissait significativement de la marche dans 26 cas. Deux patients (1,29%) avaient des antécédents d’hospitalisation pour une insuffisance cardiaque. La durée médiane d’évolution du diabète était de 2 ans (IIQ: 1-9) et l’équilibre glycémique des 3 derniers mois, exploré par l’hémoglobine glyquée était mauvais dans 138 cas (89,3%). La dyspnée d’effort était le signe fonctionnel le plus représenté avec 28 cas (18,6%) dont 22 cas du stade 2-3 de la New York Heart Association (NYHA), suivie des palpitations dans 7 cas (4,52%).

### Les anomalies électrocardiographiques

Les troubles de la repolarisation étaient l’anomalie électrocardiographique la plus fréquente dans 48 cas (31%). Il s’agissait de l’ischémie sous épicardique dans 35 cas (22,5%), d’ondes Q de nécrose dans 8 cas (5%) et de lésions sous endocardiques dans 5 cas (3,2%). Les troubles du rythme était dominés par les extrasystoles atriales (ESA) dans 25 cas (16,12%).On notait par contre une proportion faible d’hypertrophie ventriculaire gauche (HVG) dans 5 cas (3,2%) (Tableau 1).

**Tableau 1:** anomalies électrocardiographiques des 155 patients présentant un diabète de type 2 reçus dans le Département de médecine du CHUSS de Bobo d’Avril à Septembre 2014

Données	N (%)
Tachycardie sinusale	16 (10,32)
Bradycardie sinusale	11 (7,1)
ESA	25 (16,12)
ESV	1 (0,65)
HAG	22 (14,19)
HAD	1 (0,65)
HAG+HAD	2 (1,29)
BBG	2 (1,29)
BBD	1 (0,65)
HVG	5 (3,2)
Onde Q de nécrose	8 (5)
ISE	35 (22,5)
LSE	5 (3,2)
QTc allongé	9 (5,8)

ECG = Electrocardiographie ESA = Extrasystole atriale; ESV = Extrasystole Ventriculaire; HAG = Hypertrophie atriale gauche; HAD = Hypertrophie Atriale droite ; BBG = Bloc de Branche Gauche; BBD = Bloc de Branche Droit; HVG = Hypertrophie ventriculaire gauche; ISE = Ischémie sous épicardique; LES = Lésion sous endocardique

### Les anomalies échocardiographiques

L’HVG était l’anomalie la plus fréquente dans 32 cas (20,64%) suivie du remodelage ventriculaire gauche dans 22 cas (14,2%). L’oreillette gauche (OG) était dilatée dans 22 cas (14,2%), une dilatation du ventricule gauche dans 2 cas (1,3%) et une altération de la fraction d’éjection du ventricule gauche (FEVG) était notée dans 6 cas (3,9%). Les pressions de remplissage ventriculaire gauche étaient augmentées dans 22 cas (14,2%) (Tableau 2).

**Tableau 2:** Caractéristiques échocardiographiques des 155 patients présentant un diabète de type 2 reçus dans le département de médecine du CHUSS de Bobo d’Avril à Septembre 2014

Données	N (%) Médiane [IIQ]
ETT normale	90 (58)
DTDVG	44 [47-51]
DTSVG	26 [30-33]
SIVd	8 [10-11]
PPd	8 [9-10]
FEVG	64 [69-74]
Dilatation VG	2 [1,3]
Anomalies des cinétiques segmentaires	14 [9,03]
HVG	32 (20,64)
Remodelage VG	22 (14,19)
Dilatation OG	22 (14,19)
PRVG élevées	22 (14,19)
FEVG altérée	6 (3,87)
Dilatation de l’aorte initiale	3 (1,93)

ETT= Echocardiographie Transthoracique; VG = Ventricule gauche; DTDVG = Diamètre Télédiastolique VG (mm); DTSVG = Diamètre Télésystolique VG (mm); SIVd: Epaisseur septal en diastole (mm); PPd = Epaisseur paroi postérieure en diastole (mm); HVG = Hypertrophie ventriculaire gauche; OG = Oreillette gauche, PRVG=Pression de remplissage VG; FEVG = Fraction d’éjection VG

### Les entités nosologiques observées

Un total de 50 patients (32,2%) présentait une atteinte cardiaque. Les entités nosologiques étaient représentées par la cardiomyopathie hypertensive dans 27 cas (54%), la cardiopathie ischémique dans 19 cas (38%), la cardiomyopathie dilatée dans 2 cas (4%) et la cardiomyopathie diabétique dans 2 cas (4%). L’insuffisance cardiaque était notée dans 22 cas (44%) indépendamment de l’atteinte cardiaque présentée.

## Discussion

### Anomalies ECG

Notre travail montre que les anomalies ECG sont fréquentes dans la population de diabétiques de type 2. Bien que certaines anomalies soient d’allures bénignes, d’autres sont fortement prémonitoires d’évènements cardiovasculaires graves comme l’infarctus du myocarde et/ou l’insuffisance cardiaque dans cette population. La forte prévalence de l’atteinte ischémique (31% dans notre série), retrouvée dans d’autres séries Africaines [5, 10, 11] devrait placer le diabétique de type 2 dans une logique de prévention secondaire par la mise en œuvre anticipée et renforcée de mesures hygiéno-diététiques et thérapeutiques avec un contrôle plus strict des facteurs de risque associés à la prescription d’agents médicamenteux qui ont déjà prouvé leur efficacité dans le domaine de la prévention [12]. La valeur prédictive de l’ECG de repos est faible. En dépit d’un ECG de repos normal, certains sujets diabétiques asymptomatiques peuvent avoir une coronaropathie sévère pluritronculaire.

Néanmoins la réalisation de l’ECG de repos est recommandée de façon annuelle chez le diabétique de type 2 en ce sens qu’une anomalie de l’ECG de repos possède une valeur pronostique incontestable [13]. En effet, la découverte d’une anomalie évocatrice d’une ischémie myocardique, telle qu’une onde Q de nécrose ou bien une onde T d’ischémie sous épicardique, notée dans au moins trois dérivations contiguës doit motiver une exploration coronarographique de première intention et cela d’autant plus que l’anomalie apparait dans le suivi d’un diabétique dont l’ECG était précédemment normal. Dans notre contexte de travail, l’accès à cet examen simple annuellement dans le suivi du diabétique est difficile de cause multifactorielle (pauvreté, non-disponibilité et/ou rupture de consommable…). En outre, l’ECG d’effort à la recherche de l’ischémie myocardique silencieuse est inexistant. Des efforts devraient être faits pour améliorer le dépistage de la maladie coronaire dans notre population de diabétique afin de mener des actions de préventions secondaires.

### Anomalies échocardiographiques

Dans notre série, les anomalies échocardiographiques étaient dominées par l’HVG avec une fréquence de 20,64%. Cette proportion était comparable à celle rapportée par Nguyen et Barthelemy respectivement de 24,1% et 34,2% [14, 15]. Cette forte prévalence de l’HVG s’expliquerait par une association au diabète d’une forte prévalence de l’HTA. En effet, cette association était rapportée dans notre série dans 67,09% des cas superposable à celles rapportées par Nguyen *et al.*, Barthelemy *et al.* respectivement de 74% et 75% [14, 15]. Cette association HVG/HTA définie la cardiopathie hypertensive retrouvée dans 25,96% des hypertendues diabétiques. Elle serait la conséquence d’une insuffisance dans le diagnostic précoce et la prise en charge adéquate de l’HTA. En effet, dans notre série 21,3% des hypertendus ont été diagnostiqués au cours de l’étude et 76,5% des hypertendus n’atteignaient pas les objectifs tensionnels sous traitement. Cette situation interpelle une meilleure coordination entre diabétologue et cardiologue pour une optimisation de prise en charge des diabétiques de type 2. La mise en place de programme d’éducation thérapeutique au profit des diabétiques serait un meilleur atout pour une optimisation de leur prise en charge.

L’évaluation des pressions de remplissage du VG constitue une étape importante dans la réalisation de l’échocardiographie chez le diabétique. Elevées en dehors de l’HTA et/ou de la cardiopathie ischémique, elles permettent de définir la cardiomyopathie diabétique. Dans notre série, la cardiomyopathie diabétique était retrouvée dans 4% des cas. Notre taux est inférieur à ceux rapportés par les séries européennes et américaines entre 15 et 25% [15-17]. Ce fait tient au caractère relativement récent de son individualisation et à la difficulté de son diagnostic. En effet il est bien difficile de faire la part de ce qu’il convient d’imputer au vieillissement, à la coronaropathie ou à l’HTA. La définition repose aussi sur la dysfonction diastolique qui est un paramètre échocardiographique cependant peu accessible à nos populations. Néanmoins, son dépistage devrait être systématique chez les patients diabétiques par la réalisation d’une échocardiographie annuelle avec évaluation des pressions de remplissage du VG car la dysfonction diastolique précède la dysfonction systolique et toutes deux sont de pronostic sombre pour le patient diabétique [4].

L’altération de la fonction systolique du ventricule gauche était notée dans 3,9% des cas dans notre série. Elle était associée à l’élévation des pressions de remplissage retrouvées dans 14,2% des cas. Cette association définissait l’insuffisance cardiaque qui était retrouvée dans 22 cas soit 44% des entités nosologiques. Globalement, dans les différents essais et registres, la prévalence de l’insuffisance cardiaque chez les diabétiques varie entre 20 et 40% indépendamment de la fonction systolique [17]. Ce fait tient à l’intrication des autres facteurs d’insuffisance cardiaque, notamment l’HTA et l’insuffisance coronarienne [4]. Le pronostic de l’insuffisance cardiaque, quelle qu’en soit la cause, est sombre chez les patients diabétiques. Un dépistage plus précoce, permettant de mettre en place des mesures de prévention et d’adapter la prise en charge de ces patients, est indispensable. Cette démarche doit s’appuyer sur un renforcement de la collaboration entre diabétologues et cardiologues. L’ETT de repos n’a pas d’indication dans le dépistage de l’ischémie silencieuse. L’échocardiographie de stress reste l’examen de deuxième intention chez les patients incapables de réaliser un effort ou présentant un test d’effort douteux [18]. Le plateau technique de notre structure hospitalière ne permet pas encore la réalisation de cet examen.

Malgré nos moyens d’exploration limités, le bilan annuel du diabétique asymptomatique doit comporter un examen clinique attentif (recherche d’un angor d’effort, d’une claudication, d’équivalents angineux, de dyspnée d’effort) et un ECG de repos. Associés au bilan lipidique et à l’évaluation de la fonction rénale, on devrait insister sur l’évaluation du risque cardiovasculaire global de chaque diabétique par le score de Framingham et d’orienter ainsi la stratégie de dépistage. Ce minimum devrait permettre aussi d’identifier les plus à risque sur la foi des données électrocardiographiques qui présentent déjà une très probable coronaropathie (31% dans notre série).

## Conclusion

Les anomalies électrocardiographiques et échocardiographiques sont fréquentes dans la population de diabétique de type 2 au CHUSS de Bobo-Dioulasso. Elles sont dominées par l’ischémie myocardique asymptomatique et l’hypertrophie ventriculaire gauche. Les insuffisances cardiaques indépendamment de l’atteinte cardiaque sont fréquentes et dont le pronostic dans cette population reste sombre. Une meilleure collaboration entre cardiologues et diabétologues et la mise en place d’un plateau technique adéquat de dépistage et de traitement de l’ischémie myocardique serait un préalable pour une meilleure prise en charge du risque cardiaque dans notre population de diabétique de type 2.

### Etat des connaissances actuelles sur le sujet

- La première cause de décès chez les diabétiques est due aux atteintes cardio-vasculaires;
- Le diagnostic de ses atteintes cardio-vasculaires fait appel à des examens complémentaires dont les plus simples demeurent l’ECG et l’échocardiographie;
- Aucune donnée sur la question n’est disponible pour le CHUSS de Bobo-Dioulasso.

### Contribution de notre étude à la connaissance

- La prévalence des atteintes cardiaques du diabète est connue à Bobo-Dioulasso;
- L’amélioration du dépistage des atteintes cardiaques chez le diabétique nécessite une amélioration du plateau technique en particulier la détection de l’ischémie myocardique silencieuse;
- Une meilleure collaboration entre cardiologues et diabétologue est établie.

## Conflits d’intérêts

Les auteurs ne déclarent aucun conflit d'intérêts.
